# Mobilization of pro-inflammatory lipids in obese Plscr3-deficient mice

**DOI:** 10.1186/gb-2007-8-3-r38

**Published:** 2007-03-13

**Authors:** David M Mutch, Grace O'Maille, William R Wikoff, Therese Wiedmer, Peter J Sims, Gary Siuzdak

**Affiliations:** 1 The Scripps Research Institute, Department of Molecular and Experimental Medicine, North Torrey Pines Road, La Jolla, CA 92037, USA; 2 The Scripps Research Institute, Department of Molecular Biology and the Center for Mass Spectrometry, North Torrey Pines Road, La Jolla, CA 92037, USA; 3 Current address: INSERM U755 Nutriomique, Paris, F-75004 France; Pierre and Marie Curie - Paris 6 University, Faculty of Medicine, Les Cordeliers, 75004 Paris, France; 4 Current address: Department of Pathology and Laboratory of Medicine, University of Rochester Medical Center, Rochester, NY 14642, USA

## Abstract

**Background:**

The obesity epidemic has prompted the search for candidate genes capable of influencing adipose function. One such candidate, that encoding phospholipid scramblase 3 (PLSCR3), was recently identified, as genetic deletion of it led to lipid accumulation in abdominal fat pads and changes characteristic of metabolic syndrome. Because adipose tissue is increasingly recognized as an endocrine organ, capable of releasing small molecules that modulate disparate physiological processes, we examined the plasma from wild-type, *Plscr1*-/-, *Plscr3*-/- and *Plscr1&3*-/- mice. Using an untargeted comprehensive metabolite profiling approach coupled with targeted gene expression analyses, the perturbed biochemistry and functional redundancy of PLSCR proteins was assessed.

**Results:**

Nineteen metabolites were differentially and similarly regulated in both *Plscr3*-/- and *Plscr1&3*-/- animals, of which five were characterized from accurate mass, tandem mass spectrometry data and their correlation to the Metlin database as lysophosphatidylcholine (LPC) species enriched with C16:1, C18:1, C20:3, C20:5 and C22:5 fatty acids. No significant changes in the plasma metabolome were detected upon elimination of PLSCR1, indicating that increases in pro-inflammatory lipids are specifically associated with the obese state of *Plscr3*-deficient animals. Correspondingly, increases in white adipose lipogenic gene expression confirm a role for PLSCR3 in adipose lipid metabolism.

**Conclusion:**

The untargeted profiling of circulating metabolites suggests no detectable functional redundancies between PLSCR proteins; however, this approach simultaneously identified previously unrecognized lipid metabolites that suggest a novel molecular link between obesity, inflammation and the downstream consequences associated with PLSCR3-deficiency.

## Abstract 2

Metabolic profiling of mice deficient in phospholipid scramblase 3 reveals a possible molecular link between obesity and inflammation.

## Background

Despite the overt recognition of the taxing effects of obesity on both medical and social programs throughout the world, the estimated 300 million adults currently considered clinically obese in addition to the universal increase in childhood obesity indicates we are still succumbing to this global epidemic. Indeed, the poorly understood gene-environment interactions have revealed the complexity of this metabolic disease; however, with each passing year an increasing number of genetic candidates are discovered that help to further unravel the perturbed metabolism underlying the obese phenotype [1,2]. Recently, phospholipid scramblase (*Plscr*) 3 was identified as a genetic candidate capable of influencing adipose function and, ultimately, the obese phenotype. Mice deficient in PLSCR3 were found to accumulate lipid in abdominal fat pads and were characterized with insulin resistance, dyslipidemia, and glucose intolerance, classic tell-tale markers for metabolic syndrome [3]. While these observations suggest a role for PLSCR3 in adipose function, much work remains if the obese phenotype and the downstream consequences stemming from a dysfunctional PLSCR3 are to be understood.

PLSCR3 is one of four structurally related members (termed PLSCR1 through 4) in the phospholipid scramblase family [4]. The first of these plasma membrane proteins to be cloned and characterized (PLSCR1) implicated this protein family in the trans-bilayer migration of membrane phospholipids in platelets, erythrocytes, and other cell types in response to an elevation in intracellular calcium. As such, these proteins were thought to have roles in platelet procoagulent activity, cell injury by complement, and apoptosis. Since their initial discovery, phospholipid scramblases are hypothesized to have a more complex biology than previously thought. Studies aimed at defining the biological functions of *Plscr1*, the most widely studied member of the phospholipid scramblase family, have demonstrated that it is transcriptionally up-regulated by interferon-α and other factors [5,6], and that a portion of the newly synthesized PLSCR1 protein can translocate into the cell's nucleus and interact with genomic DNA, suggesting it has a potential role in the regulation of gene expression [7,8].

The development of murine models deficient in PLSCR proteins provides a means to elucidate the biochemistry underlying *Plscr3*-mediated obesity. As previously observed with members of a protein family, a degree of redundancy exists in order for an organism to maintain physiological homeostasis and preserve the full gamut of biological functions required for survival [9,10]. With regards to the phospholipid scramblases, adipocytes accumulate neutral lipid in *Plscr3*-deficient mice; however, *Plscr1*-deficient animals also have a small increase in adipose lipid and the *Plscr1&3*-deficient mice have an even greater accumulation of lipid than the *Plscr3*-null mice [3]. This would suggest that, to some extent, a redundancy in the adipose functions of these two proteins may exist.

To begin to unravel the perturbed biochemistry associated with *Plscr3 *deficiency, we employed comprehensive metabolite screening technologies to determine whether the biological abnormalities stemming from the lack of PLSCR3 protein are reflected in plasma. As metabolites represent a metabolic endpoint of gene and protein function, their analysis provides insight into the cellular function of genetically modified mice [11,12]. As such, untargeted metabolomics offers a powerful method to further define the obese phenotype in organisms characterized by genetic modifications [13,14]. Furthermore, an additional inherent advantage of untargeted metabolomics (which can also be extended to alternative functional genomic strategies) versus a more targeted analysis (that is, the lipidome [15]) is its ability to generate novel hypotheses through the identification of previously unrecognized signaling pathways [2]. As demonstrated in the present manuscript, the characterized metabolites identified in animals lacking *Plscr3 *suggest a novel molecular link between the chronic low-level inflammation characteristic of an obese state and the heightened downstream risk of cardiovascular disease.

## Results and discussion

**Figure 1:**
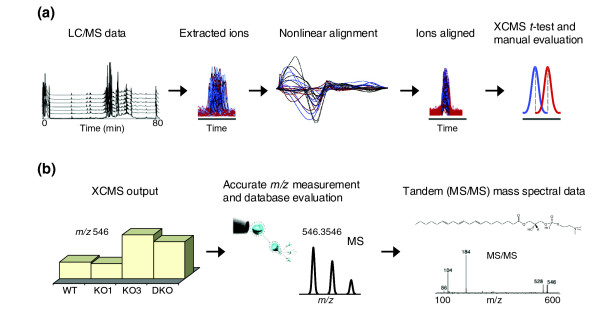
Graphical depiction of workflow for the identification of metabolites. **(a) **Mass spectral data collection and processing using XCMS. LC/MS data were first analyzed with XCMS software to produce a list of metabolite features, where each feature is defined by both a specific retention time and *m/z *value. The XCMS software then applies a non-linear retention time correction to align the same metabolite features found in different biological samples. The final XCMS output lists the *t*-test results based on the intensity variations of common metabolite features found among the defined sample classes. **(b) **Metabolite selection, characterization and identification. Metabolite features meeting statistical criteria for significance, based on XCMS processing, were further characterized by accurate mass measurement, identified using mass spectral databases such as the Metlin database and the LIPID MAPS database, and further confirmed by tandem (MS/MS) mass spectral data.

Plasma from male mice of each genotype (*n *= 4) were obtained, separated into two groups of two, and analyzed in triplicate at two separate run dates. As such, only metabolites that were consistently identified in all four animals of a specific genotype, irrespective of date of analysis, were considered 'true' peaks. Using XCMS software, we were able to confidently examine the data generated in the multiple analyses by first performing nonlinear alignment compensating for minor differences in ion retention times between the runs, and then identifying and matching peaks for further analysis (Figure 1). In this regard, correction of retention times permits the relative metabolite ion intensities to be statistically compared between the various genotypes in order to identify unique and/or shared ions associated with deficiencies in PLSCR proteins. For this to be accomplished with confidence, a number of criteria were used prior to considering an ion as significantly different between the various phenotypes. Initially, an intensity threshold was selected to ensure that ions could be subsequently enriched and analyzed for their accurate mass by electrospray ionization coupled with time of flight analysis (ESI-TOF). Subsequent to this analysis, ions were only deemed significantly different between the various genotypes if they had a *p *value ≤ 0.01. Furthermore, only those metabolites simultaneously identified in both a single knock-out model and the double knock-out model (that is, KO1 and DKO, or KO3 and DKO) were considered as 'candidate metabolites'. This approach meant that all metabolites discussed in this manuscript were identified in eight mice (each in triplicate liquid chromatography (LC) runs), thereby strengthening the biological relevance and statistical significance of our results. Finally, data generated on the individual ions were manually inspected to validate the changes and identify the most important molecules for future isolation.

**Figure 2:**
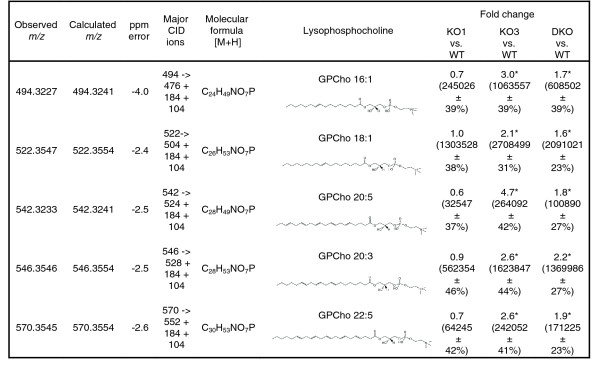
Exact masses and chemical structures of metabolites characterized with the Metlin database. The significance of fold changes between the KO1 versus WT, KO3 versus WT, and DKO versus WT are indicated with an asterisk where *p *< 0.005. The significance for all 5 metabolites is further increased to p < 0.00001 when comparing KO3 and DKO to KO1 and WT. Average intensity values ± relative standard deviation values are indicated in parentheses below the fold changes, and demonstrate the large difference in metabolite abundance between the five LPC molecules. Structures were obtained from the LipidMaps website [34].

All possible comparisons were performed, with the goal of identifying metabolites both unique to a specific genotype and shared between two or more genotypes. XCMS detected over 10,000 peaks across the 48 LC/mass spectrometry (MS) runs performed (corresponding to 16 mice, each run in triplicate; see Additional data file 1 for all raw data); however, to minimize spurious results, we considered only those peaks that were consistently identified in a genotype (that is, found in 12 LC/MS runs). The comparison between wild type (WT) and the three knock-out models did not identify common metabolites amongst the various mutant models, suggesting that *Plscr1 *and *Plscr3 *affect the plasma metabolome differently. Pair-wise comparisons (WT and KO1 versus KO3 and DKO, WT and KO3 versus KO1 and DKO, and WT and DKO versus KO1 and KO3) revealed that eliminating *Plscr3 *gave rise to an increase in the abundance of metabolites common to both KO3 and DKO models. This was not unexpected, as DKO animals have a phenotype similar to that of KO3 animals. The XCMS software identified 19 metabolites that were significantly different from WT and KO1 mice (Additional data file 2), of which 5 were characterized using the Metlin database (Figure 2) [16]. While our initial statistical cut-off for significance was arbitrarily set at a *p *value of 0.01, it is noteworthy to mention that, for all 19 identified metabolites, the *p *values were less than 0.0005, and the 5 characterized metabolites were of even greater statistical significance (*p *≤ 0.00001). It is expected that the 14 currently non-identifiable metabolites, once characterized and incorporated into public databases by the greater metabolomic community, will provide further information into the dysfunctional biology underlying the *Plscr3*-/- mouse. The five identifiable metabolites, with exact masses (m/z) of 494.3241, 522.3554, 542.3241, 546.3570, and 570.3543, correspond to lysophosphatidylcholine (LPC) species containing palmitoleic (C16:1n-7; PO), oleic (C18:1n-9; OA), dihomo-γ-linolenic (C20:3n-6; DGLA), eicosapentaenoic (C20:5n-3; EPA) and osbond (C22:5n-6; ObA) acids, respectively (Figure 2). Changes in all 19 metabolites were uniquely associated with *Plscr3 *deficiency, since these metabolites did not vary in the KO1 model. By contrast, we did not observe significant differences in metabolite profiles between the WT and KO1 animals, which was further reinforced by revealing no common, significantly modulated metabolites between the KO1 and DKO models.

Increasing evidence has positioned adipose tissue as not merely a reservoir for lipid storage, but also as a major endocrine and secretory organ. The recognition that obesity is characterized by chronic mild inflammation led to the discovery of factors, termed adipokines, that are released from adipose tissue and critical in regulating such physiological processes as inflammation, lipid metabolism, insulin sensitivity, angiogenesis, and eating behavior [17,18]. Furthermore, several factors have been shown to regulate atherogenic processes, such as hypertension and vascular remodeling [19,20]. It is interesting to find that plasma from *Plscr3*-deficient mice show increases in pro-inflammatory LPC molecules that have been linked to certain pathophysiological conditions, including atherosclerosis, cancer and rheumatoid arthritis [21-23]. While these bioactive lysophospholipids have not been previously described in obesity, their identification suggests a novel pro-inflammatory class linking obesity and atherosclerosis that merits further examination. The characterization of normal human serum revealed that four LPC metabolites (LPC containing 16:0, 18:0, 18:1, or 18:2 fatty acids) are among the most abundant circulating metabolites found [24]. The genetic deletion of murine *Plscr3 *led to significant changes in only one of these highly abundant LPC species (18:1). Additionally, as changes in the abundance of other lysophospholipid classes were not observed, this indicates that increases in LPC species are specific to *Plscr3 *deficiency. Although to our knowledge LPC molecules have not been previously examined in obese models (either rodent or human), this finding may be a general characteristic of the obese state and requires confirmation in other models of obesity.

Conformational and positional characterization of the acyl portion of lipid metabolites was not performed; therefore, the discussion herein is based on the previous reports of the most common fatty acids produced in eukaryotes [25]. The LPC species found to be most abundant in plasma was oleoyl-LPC (Figure 2). Due to this abundance, we were able to further confirm the presence of this molecule by converting it to the corresponding fatty acid methyl ester, followed by gas chromatography (GC)/MS analysis. After production of the methyl esters, the GC/MS experiment confirmed the presence of the expected fatty acid C18:1 (C_18_H_34_O_2_) in an LPC molecule. More specifically, the hypothesized molecular ion for the 18:1 methyl ester (m/z 296, M+H) and the fragmentation pattern were found, which matched the correct model spectrum in the NIST 2002 spectral database. Both palmitoleic (C16:1n7) and oleic acids (C18:1n9) are synthesized via stearoyl-CoA desaturase (SCD1), the rate-limiting enzyme regulating the introduction of a *cis*-double bond in the Δ9 position of its fatty acyl-CoA substrates palmitate (C16:0) and stearate (C18:0). SCD1 has a pivotal role in whole-body lipid metabolism, as exemplified by the finding that Scd1-deficient mice are resistant to diet-induced obesity [26]. However, the physiological complexity underlying the obese state suggests that interpreting plasma metabolite profiles has the power to identify biomarkers without indicating their originating tissue source.

**Figure 3:**
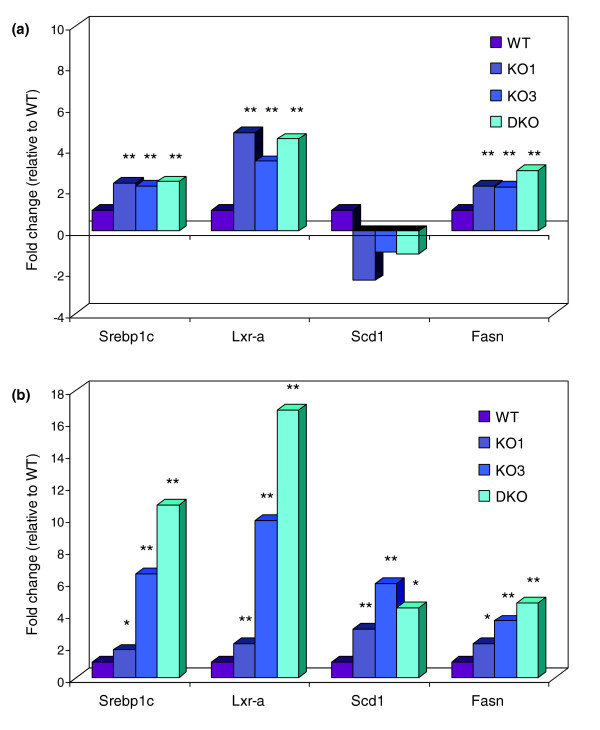
Assessing gene expression in **(a) **liver and **(b) **WAT. A subset of genes under the transcriptional regulation of Srebp-1c were examined by real-time RT-PCR and, in WAT, were more significantly and strongly up-regulated in the absence of Plscr3. As the goal was to address genotype differences and not individual animal variation, total RNA from three animals/genotype was pooled and technical triplicates were run. *p < 0.01, **p < 0.001.

Thus, to begin to elucidate the tissue responsible for altering lipid metabolism in *Plscr3*-/- mice, the expression of a subset of lipogenic genes (those encoding *Ppar*-γ, *Lxr*-α, *Srebp-1c*, *Fasn*, and *Scd1*) was examined in the liver and white adipose tissue (WAT) of the various genotypes. While a similar profile for *Ppar*-γ expression was observed in both tissues - WAT (KO1, 2.0-fold; KO3, 5.5-fold; DKO, 9.4-fold) and liver (KO1, 4.4-fold; KO3, 12.1-fold; DKO, 39.5-fold) - the expression pattern for the remaining genes were tissue-specific. Hepatic gene expression in the three knock-out models indicated similar fold-increases in *Lxr*-α, *Srebp-1c*, and *Fasn*, with the exception of *Scd1*, which was not significantly changed in the liver (Figure 3a). In contrast, gene expression in WAT revealed marked differences between the various genotypes (Figure 3b). For all genes examined, induction was minimal and less significant in KO1 mice, whereas changes in KO3 and DKO mice were highly significant and of greater importance (*p *≤ 0.001). Of particular interest was the expression of *Lxr*-α and *Srebp*-1c, transcriptional regulators of *Fasn *and *Scd1*. KO1 animals had only a slight 1.7-fold increase in *Srebp-1c *expression, while KO3 and DKO mice had far more prominent increases of 6.5- and 10.8-fold, respectively. Similar changes were seen with *Lxr*-α (KO1, 2.1-fold; KO3, 9.8-fold; DKO, 16.8-fold), *Fasn *(KO1, 2.1-fold; KO3, 3.6-fold; DKO, 4.7-fold) and *Scd1 *expression (KO1, 3.0-fold; KO3, 5.9-fold; DKO, 4.4-fold). As *Plscr3 *expression is very low in liver [27] and *Plscr1 *expression is nearly absent in WAT [3], the transcriptional and lipid metabolite changes observed with *Plscr3 *deficiency would suggest they originate from the WAT and may be directly related to the SREBP-1c-mediated transcriptional cascade. Indeed, if the plasma lipid profile reflected hepatic transcriptional events, one could hypothesize that all genotypes would have similar changes in plasma lipids; however, since this was not the case, we propose that the differential plasma lipid profile arises from transcriptional events occurring in WAT.

As described above, *Plscr3*-/- mice have an increase in Δ9-desaturase expression similar to that previously noted in various obese models and animals fed diets promoting weight gain (that is, obesity-inducing diets increased *Scd1 *expression and activity, while diets reducing adiposity decrease it) [11]. The identification of DGLA, EPA, and ObA fatty acid species may reflect additional modulation of adipose Δ5 and Δ6 desaturase enzyme activities, as both enzymes have roles in the metabolism of long-chain polyunsaturated fatty acid (LC-PUFA) species [28,29]; however, in the absence of adipose metabolite data this hypothesis remains to be proven. It has recently been proposed by Das [30] that defects in Δ5 and Δ6 desaturases may play a role in the development of insulin resistance by reducing the synthesis of beneficial LC-PUFA arachidonic acid (AA) and docosahexaenoic acid (DHA). *Plscr3*-/- mice are insulin resistant [3] and the increase in 22:5-containing LPC would further support Das's hypothesis, as increases in the abundance of 22:5 fatty acids are considered an indicator of DHA deficiency [31]. These findings position *Plscr3 *as a regulator of adipose lipid metabolism.

Of additional interest, both DGLA and EPA are purported to have anti-inflammatory properties via their conversion into the 1 series and 3 series of prostaglandins, respectively [32]. Although the precise contribution of ω-3 and ω-6 LC-PUFA to the inflammatory state has yet to be resolved, both classes are precursors to inflammatory eicosanoids; however, it appears that ω-3 LC-PUFA derived eicosanoids are associated with a less severe inflammatory profile than those derived from ω-6 LC-PUFA [33]. Thus, it is conceivable that plasmatic increases in LPC containing DGLA and EPA reflect altered biochemical pathways in WAT eicosanoid metabolism associated with a deficiency in *Plscr3*; however, no changes in the eicosanoids currently characterized in public databases (Metlin [16], LipidMaps [34]) were found in plasma.

## Conclusion

Plasma metabolite profiling coupled with analytical software such as XCMS provides a method by which the unique biochemical signatures and functional redundancy of related proteins can be explored. Furthermore, the ability to identify perturbations in previously unrecognized metabolic pathways reinforces the potential of untargeted metabolite profiling for generating hypotheses and new research directions. As demonstrated in the present study, *Plscr1 *and *Plscr3 *do not modulate the plasma metabolome in a similar fashion. While no changes were detected in the plasma metabolite profile upon genetic deletion of *Plscr1*, deletion of *Plscr3 *clearly modulates the plasma metabolome through the release of pro-inflammatory lipids and other, currently unidentifiable, small molecules. Although profiling plasma metabolites does not definitively unravel the molecular mechanisms underlying lipid accumulation in *Plscr3*-deficient animals, invaluable clues were provided into the perturbed physiology of these animals and will serve as indicators for future targeted experiments. Not only do LPC molecules present a potential link between obesity, inflammation, and atherogenesis, the acyl composition of LPC suggests that *Plscr3*-deficient mice may have modified desaturase enzyme activities and eicosanoid metabolism. Indeed, *Scd1 *and its transcriptional regulators (*Lxr*-α, *Srebp-1c*) are significantly up-regulated in the adipose of *Plscr3*-deficient mice. Furthermore, the accumulation of LC-PUFA precursors (DGLA, EPA, and ObA) in LPC species may suggest deficiencies in the abundance of beneficial AA and DHA fatty acids. To reinforce this notion, decreases in LC-PUFA and increases in monounsaturated species were previously reported in obese Zucker rats [35]. It is interesting to note that additional work by Wilson and colleagues [36,37] has also demonstrated the ability to discriminate the plasma metabolite profiles of lean and obese 20-week old Zucker (fa/fa) rats using metabolomics coupled with bioinformatics algorithms. While the authors found six metabolites in the positive ion data of an LC/MS analysis to be different between the two rat strains, none of these small molecules were identified [38]. Thus, while the untargeted metabolite profiling presented in this study is still in its early stages of development, the ability to identify and correlate metabolites with functionally related protein family members and provide novel and previously unrecognized insight into the perturbed metabolism stemming from dysfunctional genes positions this analytical platform as an attractive means towards understanding the fundamental biochemistry of disease states.

## Materials and methods

### Animals

*Plscr1*-/- (KO1) and *Plscr3*-/- (KO3) mice were produced by Lexicon Genetics Incorporated (The Woodlands, TX, USA) and *Plscr(1&3)*-/- (DKO) mice were produced by breeding KO1 with KO3 mice as previously described [3,8]. The genetic background of all mice was identical (C57BL6Jx129SvEvBRD) and details of their general characterization can be found elsewhere [3,39]. All mice were fed standard (approximately 5% fat) rodent chow (Harlan Teklad, Madison, WT, USA) and had access *ad libitum *to sterilized water. Mice were fasted for four hours before blood draw. Approximately 250 μl of blood was extracted by retroorbital eye bleeds from male mice of approximately 8 weeks of age (*n *= 4 per genotype). Age-matched mice were divided into 2 groups of 2 and blood was extracted on 2 separate days to minimize differences in fasting time between all 16 mice. Blood was collected into tubes containing lyophilized K_2_EDTA (Becton Dickinson, Franklin Lakes, NJ, USA) and immediately centrifuged at 800 × g for 15 minutes at 4°C to extract plasma. After collection samples were stored at -80°C prior to analysis.

### Chemicals and sample preparation

All solvents used were of HPLC grade (JT Baker, Philipsburg, NJ, USA). Metabolite extraction was performed with cold methanol as described previously [24]. Briefly, 40 μl aliquots of mouse plasma were extracted with 150 μl cold methanol, and incubated at -20°C for 20 minutes, then centrifuged to remove protein precipitate. The supernatant was dried and reconstituted in 40 μl acetonitrile/water 5/95 v/v.

### LC/MS data acquisition and analysis

The separation system used was an Agilent 1100 LC/MSD SL system equipped with HPLC (Agilent, Santa Clara, CA, USA). Triplicate runs of each sample were analyzed randomly, with a blank run between samples to prevent carryover. For each run, 5 μl of metabolite extract was injected onto the same C_18 _column (Symmetry Column, 2.1 × 100 mm, 3.5 μm, Waters (Waters, Milford, MA, USA) and eluted at a flow rate of 250 μl/minute. Elution buffers were: A, water with 0.1% formic acid; and B, acetonitrile with 0.1% formic acid. The LC/MS run time was 75 minutes, with a gradient begun at 5% B until 12 minutes, with times and percentages as follows: 20% B at 20 minutes, 90% B at 55 minutes, 95% B at 60 to 70 minutes, 5% B at 71 to 75 minutes. Mass spectral data from 100 to 1,000 *m/z *were collected in the positive ionization mode. LC/MS data were processed using the XCMS software [40]. Metabolites of interest were selected based on values of ion intensity changes and consistency between animals of the same type.

### Accurate mass and MS/MS fragmentation determination

Fractions containing the metabolites of interest were collected in subsequent HPLC separations. These fractions were then analyzed individually in positive ion mode using the Agilent ESI-TOF to obtain high accuracy mass spectral data (<4 ppm error between observed and calculated masses; Additional data file 1). Three reference masses with *m/z *at 121.0509, 319.1030, and 922.0098 were used for real time mass adjustments. MS/MS data were collected using a linear ion trap (Thermo, Waltham, MA, USA). Specific masses that varied significantly between WT versus KO3 and DKO mice were targeted for fragmentation. MS/MS conditions were as follows: isolation = 3.0 amu, normalized collision energy = 35%, activation Q = 0.15 and activation time = 30.0 ms.

### GC/MS identification of lysophospholipid LPC 18:1

An experiment was designed to confirm the identification of the lysophosphocholine metabolite at m/z 522, using a fatty-acid methyl ester approach (FAME) coupled with GC/MS. The LC/MS chromatography of plasma was repeated with a larger volume of starting material (equivalent to 32 μl) and 1 minute fractions were collected by hand. This preparative chromatography was repeated once, with pooling of the equivalent fractions to increase the final yield. The fraction between 44.5 and 45.5 minutes was expected to contain the metabolite with m/z 522.3547, and this was confirmed by locating the 522 mass by direct injection of 4% of this fraction into the ESI-TOF spectrometer. The sample was then converted to fatty acid methyl esters, dried, and 200 μl of a 3N HCl methanol solution was added and incubated in a 100°C oven for 45 minutes. Hexane (400 μl) was added, and the solution was dried and reconstituted in dichoromethane (DCM). The GC/MS column was a HP5-MS (J&W Scientific/Agilent, Santa Clara, CA, USA) with the following characteristics: length = 30 m, ID = 0.25 mm, and film = 0.25 μm. The mass spectrometer was an Agilent 5973 with an injector port temperature of 290°C and a transfer line temperature of 280°C. The flow rate was 1.2 ml/minute with a total run time of 27.5 minutes. The temperature program was 50°C for 5 minutes, followed by a gradient of 20°C/minute to 300°C, followed by a hold at 300°C for 10 minutes. The injection volume was 2.5 μl with no split. MS/MS data were collected as described in the preceding section.

### Semi-quantitative real-time RT-PCR

Total RNA from three animals/genotype was pooled for the liver and white adipose tissue. Reverse transcription was performed with 1 μg of total RNA using the Advantage RT-PCR kit (Clontech, Mountain View, CA, USA) and random hexamer primers. Sybr^® ^green primers were designed (Additional data file 3) and validated for target specificity and amplification efficiency. RT-PCR amplification was performed using a BioRad iCycler (BioRad, Hercules, CA, USA) with the following thermal cycling conditions: 2 minutes at 50°C, 10 minutes at 95°C, followed by 40 cycles of 95°C for 15 s and 60°C for 1 minute for denaturation, annealing, and elongation. All samples were performed in (technical) triplicate and data were normalized to glyceraldehyde-3-phosphate dehydrogenase. A two-tailed, homoscedastic Student's *t*-test (α = 0.01) was used to confirm differences in gene expression in pair-wise analysis (that is, genotypes compared to WT).

## Additional data files

The following additional data are available with the online version of this paper. Additional data file 1 is a table containing the raw dataset from XCMS processing, where 'name' denotes feature or peak name, 'M' denotes m/z, 'T' denotes retention time in units of seconds, 'mzme' denotes median m/z value, 'mzmin' denotes minimum m/z value, 'mzmax' denotes maximum m/z value, 'rtmed' denotes median retention time in seconds, 'rtmin' denotes minimum retention time in seconds, 'rtmax' denotes maximum retention time in seconds, 'WT' denotes wild-type animal intensity area values, 'KO3' denotes Plscr3-/- animal intensity area values, 'KO1' denotes Plscr1-/- animal intensity area values, 'DKO' denotes double knock-out animals animal intensity area values, and the letters a, b, and c following Mouse strain_Mouse Number denote the replicated experimental runs. Additional data file 2 lists the 19 metabolites associated with PLSCR3 deficiency. Additional data file 3 is a table containing sequence information for real-time RT-PCR primers.

## Supplementary Material

Additional data file 1 Name, feature or peak name; M, m/z; T, retention time in units of seconds; mzme, median m/z value; mzmin, minimum m/z value; mzmax, maximum m/z value; rtmed, median retention time in seconds; rtmin, minimum retention time in seconds; rtmax, maximum retention time in seconds; WT, wild-type animal intensity area values; KO3, Plscr3-/- animal intensity area values; KO1, Plscr1-/- animal intensity area values; DKO, double knock-out animals animal intensity area values; the letters a, b, and c following Mouse strain_Mouse Number denote the replicated experimental runs. Click here for file

Additional data file 2 The 19 metabolites associated with PLSCR3 deficiency. Click here for file

Additional data file 3 Sequence information for real-time RT-PCR primers. Click here for file
