# Supplementary material for: Mobilization of pro-inflammatory lipids in obese Plscr3-deficient mice
Source: Genome Biol. 2007 Mar 13;8(3):R38. doi: 10.1186/gb-2007-8-3-r38 (PMC1868938; doi:10.1186/gb-2007-8-3-r38)
Supplement: Additional data file 3 — Sequence information for real-time RT-PCR primers. [file gb-2007-8-3-r38-S3.doc]

| **Gene Symbol** | **Genebank**  **Accession #** | **5’ primer** | **3’ primer** |
| --- | --- | --- | --- |
| **Gapdh** | NM_008084 | caatgaatacggctacagcaaca | gtggtccagggtttcttactcct |
| **Plscr1** | NM_011636 | ctgggtatccccctccgtat | gtatggggtaagcagcatggtc |
| **Plscr3** | NM_023564.2 | caagactcccctcctgattcc | tgaaggggcatagcctttgg |
| **Scd1** | NM_009127.2 | cgagcaactgactatcatcatgc | gtggtggtggtcgtgtaagaact |
| **Ppar-γ** | NM_011146 | cttcgctgatgcactgcctat | agaatggcatctctgtgtcaacc |
| **Lxr-α** | [NM_013839.1](http://www.ncbi.nlm.nih.gov/entrez/query.fcgi?cmd=Retrieve&db=nucleotide&dopt=GenBank&list_uids=7305320) | gaaacgcgacagttttggtagag | cacaaggacatctcttcctggag |
| **Srebp-1c** | NM_011480.1 | gagatgtgcgaactggacacag | cactgtcttggttgttgatgagc |
| **Fasn** | NM_007988 | cagctatgaagcaattgtggatg | agtgttcgttcctcggagtgag |
